# Methods for the integration of multi-omics data: mathematical aspects

**DOI:** 10.1186/s12859-015-0857-9

**Published:** 2016-01-20

**Authors:** Matteo Bersanelli, Ettore Mosca, Daniel Remondini, Enrico Giampieri, Claudia Sala, Gastone Castellani, Luciano Milanesi

**Affiliations:** 1 Department of Physics and Astronomy, Universita’ di Bologna, Via B. Pichat 6/2, Bologna, 40127 Italy; 2 Institute of Biomedical Technologies - CNR, Via Fratelli Cervi 93, Segrate MI, 20090 Italy

**Keywords:** Omics, Multi-omics, Data integration

## Abstract

**Background:**

Methods for the integrative analysis of multi-omics data are required to draw a more complete and accurate picture of the dynamics of molecular systems. The complexity of biological systems, the technological limits, the large number of biological variables and the relatively low number of biological samples make the analysis of multi-omics datasets a non-trivial problem.

**Results and Conclusions:**

We review the most advanced strategies for integrating multi-omics datasets, focusing on mathematical and methodological aspects.

## Background

Biological functions are exploited by systems of interacting molecules and macromolecules that take part in physical and biochemical processes in structured environments. Different types of high-throughput technologies allow us to collect information on the molecular components of biological systems. Each of such technologies (e.g. nucleotide sequencing, DNA-chips and protein mass spectrometry) is designed to simultaneously collect a large set of molecular data of a specific kind: e.g. nucleotide sequences, gene expression and protein abundances. Therefore, in order to draw a more comprehensive view of biological processes, experimental data made on different layers have to be integrated and analyzed. The complexity of biological systems, the technological limits, the large number of biological variables and the relatively low number of biological samples make integrative analyses a challenging issue. Hence, the development of methods for the integrative analysis of multi-layer datasets is one of the most relevant problems computational scientists are addressing nowadays.

A few reviews exist on this topic. For example, Berger et al. [1] described integrative approaches in one of the sections of their work, which is also focused on tools for the analysis of single omics layers, while Kristensen et al. [2] presented objectives, methods and computational tools of integrative genomics, with a particular focus on the applications related to cancer research. Conversely, we would like to focus on mathematical aspects and illustrate the solutions found to the problem of multi-omics data integration.

The classification of the approaches presented in the literature as multi-omics methods is a non-trivial task for at least three reasons. First, most of the computational approaches developed so far are pipelines of analysis that apply several methods to carry out a sequence of tasks; therefore, different pipelines share some methods: for example, partial least squares regression is included in both Integromics [3] and sMBPLS [4]. Second, pipelines presented for addressing a particular problem can be also used, with minor modifications, to solve another problem, possibly with other types of omics. Third, several tools can be used in a supervised or unsupervised setting, according to the formulation of the problem.

## Methods

On the basis of methodological aspects, we will consider two main criteria. The first is whether the approach uses graphs to model the interactions among variables. These approaches, designated as “network-based” (NB), take into account currently known (e.g. protein-protein interactions) or predicted (e.g. from correlation analysis) relationships between biological variables. In this class, graph measures (e.g. degree, connectivity, centrality) and graph algorithms (e.g. sub-network identification) are used to identify valuable biological information. Importantly, networks are used in the modeling of the cell’s intricate wiring diagram and suggest possible mechanisms of action at the basis of healthy and pathological phenotypes [5].

The second criterion is whether the approach is bayesian (BY) [6], that is, it uses a statistical model in which, starting from an *a priori* reasonable assumption about the data probability distribution, *parametric* or *non-parametric*, it is possible to compute the updated posterior probability distribution making use of the Bayes’ rule; of course the posterior distribution depends on dataset measurements [7]. In the network-based area, bayesian networks [8–10] are another promising framework for the analysis multi-omics data.

Therefore, we will arrange integrative methods in four classes: network-free non-bayesian (NF-NBY), network-free bayesian (NF-BY), network-based non-bayesian (NB-NBY) and network-based bayesian (NB-BY) methods. We will give an overview of the methods that have been proposed for the analysis of at least two different types of omics datasets and describe with more details the specific mathematical grounds. In particular, we choose to consider in detail the mathematical aspects of the most common, representative or promising methods of each category.

## Results and Discussion

### Methods overview

Mathematically, the general problem of analyzing multiple omics datasets can be formulated as the sequential or joint analysis of multiple component-by-sample matrices, possibly using other data that carry prior information on components and samples.

**Fig. 1:**
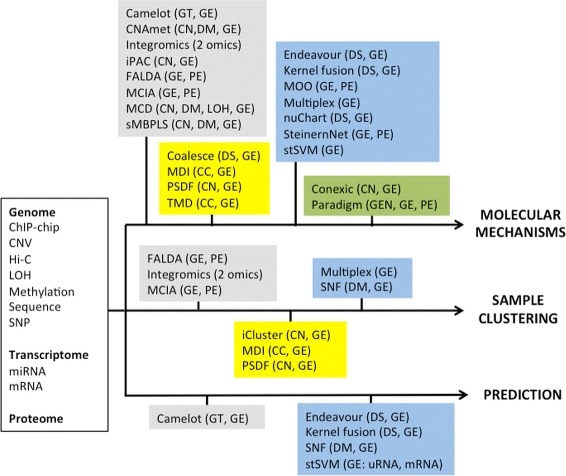
Overview of multi-omics methods. Methods are placed in boxes according to whether they make use of networks and bayesian theory; the types of omics that each method takes in input (or has been applied to in a case study) is indicated between parentheses. *Grey*: network-free, non-bayesian methods; *yellow*: network-free, bayesian methods; *blue*: network-based, non-bayesian methods; *green*: network-based bayesian methods. Abbreviations: *GEN* = genome, *CC* = ChIP-chip, *CN* = copy number variations, *DM* = DNA methylation, *DS* = DNA sequence, *Hi-C* = genome-wide data of chromosomal interactions, *LOH* = loss of heterozigosity, *GT* = genotype, *GE* = gene expression, *PE* = protein expression

The objectives of integrative analysis can be summarized into the following [2] (Fig. 1): (i) the discovery of molecular mechanisms; (ii) the clustering of samples (e.g. individuals); (iii) the prediction of an outcome, such as survival or efficacy of therapy. Most of the methods are developed for the first and second objectives, while less methods carry out prediction.

Integrative approaches can be more or less stringent on the types of omics considered in input: some methods are designed to analyze a specific combination of datasets, while others are more general. For example, Conexic [11] is tailored for DNA copy number variations (CNV) and gene expression data, while iCluster [12] can be in principle used for the analysis of any combination of omics encoded as quantitative values on the same set of samples (Table 1).

**Table 1:** Methods for the analysis of multi-omics datasets

Method	Specificity	Multi-omics approach	Implementation
Camelot [15]	Specific	Bivariate predictive regression model	NA
CNAmet [19]	Specific	Multi-omics gene-wise scores	R
FALDA [21]	General	FA + LDA of a joint matrix	NA
Integromics [3]	General	Regularized CCA, sparse PLS	R
iPAC [14]	Specific	Sequential	NA
MCD [13]	Specific	Sequential	NA
MCIA [20]	General	Multiple co-inertia analysis	R
sMBPLS [4]	General	Sparse Multi-Block PLS regression	Matlab
Coalesce [30]	Specific	Multi-omics probabilities	C ++
iCluster [12]	General	Joint Gaussian latent variable models	R
MDI [28]	General	DMA mixture models	Matlab
PSDF [29]	General	Hierarchical DMA mixture models	Matlab
TMD [27]	General	Hierarchical DMA mixture models	Matlab
Kernel Fusion [18]	General	Integration of omics-specific kernels	Matlab
Endeavour [37]	General	Integration of omics-specific ranks with order statistics	Webserver
MOO [16]	General	Sub-network extraction on MWG	R
Multiplex [38]	General	Joint analysis of multi-layered networks	NA
NuChart [35]	Specific	Analysis of a MWG	R
SNF [17]	General	Similarity network fusion	Matlab, R
SteinerNet [33]	Specific	Sub-network extraction on MWG	Webserver
stSVM [34]	Specific	MWG	R
Paradigm [51]	General	Multi-omics bayesian factor graphs	C ++
Conexic [11]	Specific	Sequential	Java

Specificity indicates whether the method was designed for a specific combination of omics (specific) or not (general). Legend: MWG = multi-weighted graph; FA = factor analysis; LDA = linear discriminant analysis; CCA = canonical correlation analysis; PLS = partial least squares; DMA = Dirichelet multinomial allocation; NA = not available

As already mentioned, a distinction can be done between *sequential* and *simultaneous* analysis of multiple layers. In the former case, the results of the analysis of one layer are refined by means of the subsequent analyses of further layers. This is the case, for example, of methods that are designed assuming a causal effect of an omics (e.g. genomics) on another (e.g. transcriptomics), like MCD [13] and iPAC [14]. The joint analysis of multiple omics can be carried out by means of models that consider each layer as a separate entity: two examples are multivariate regression [15] and multi-objective optimization [16]. Simultaneous analysis may require a preliminary step of data fusion, which usually involves objects derived from single-layer analysis: two examples are the fusion of sample-sample similarity matrices [17] and of gene-gene kernels matrices [18] calculated on different omics.

### Network-free non-bayesian (NF-NBY)

Among the approaches that have been developed for specific types of omics there are iPAC [14], MCD [13], CNAmet [19], sMB-PLS [4] and Camelot [15]. iPAC [14] is an unsupervised approach for the sequential analysis of CNV and gene expression data on the basis of a series of gene selection criteria: aberrant genes identified by the analysis of CNV are further studied by correlation analysis of gene expression in order to find the subset of aberrant genes potentially leading to a substantial shift in transcriptional programs. MCD [13] (Multiple Concerted Disruption) is another sequential approach. CNVs, loss of heterozygosity (LOH) and DNA methylations are analyzed sequentially in order to find changes in gene copy number accompanied by allelic imbalances and variations in DNA methylation resulting in gene expression differences. CNAmet [19] uses gene-wise weights calculated considering the gene expression in classes of samples with different CNVs or DNA methylation pattern; weights for CNV and DNA methylation are then linearly combined to define gene-wise statistics, whose significance is assessed by permutation analysis. In 2012 Li et al. presented the sparse Multi-Block Partial Least Squares (sMB-PLS) regression method [4] for the identification of regulatory modules from multiple omics. Common weights are found in order to maximize the covariance between summary vectors of the input matrices (CNV, DNA methylation and miRNA expression) and the summary vector of the output matrix (mRNA expression). A multi-dimensional regulatory module contains sets of regulatory factors from different layers that are likely to jointly contribute to a “gene expression factory”. Camelot [15] finds the optimal regression model for phenotype prediction (drug response) on the basis of matched genotype and gene expression data. This method suggests the molecular mechanisms that predict the phenotype under analysis.

Conversely from the methods above, Integromics [3], MCIA [20] and the approach of Liu et al. [21] are based on models of data integration that can be easily applied to different types of omics. Integromics [3] performs integrative analysis of two types of omics with the main objective of finding similarities among samples and correlation among molecular components. It uses a regularized version of canonical correlation analysis to highlight correlations between the two datasets and a sparse version of partial least squares regression that includes simultaneous variable selection in both datasets. In principle, it can be applied to any pairs of omics that can be encoded as continuous sample-by-components matrices. Multiple co-inertia analysis MCIA [20] is an exploratory data analysis method that identifies co-relationships between multiple high-dimensional datasets. Based on a covariance optimization criterion, MCIA simultaneously projects several datasets into the same dimensional space, transforming diverse sets of features onto the same scale. This analysis leads to the identification of biological markers and clusters of samples. Liu et al. [21] presented a method (shortly FALDA) based on standardization and merger of several omics (namely mRNA, miRNA and protein data) into a joint (standardized) molecule-by-sample matrix. Then, factor analysis (FA) and linear discriminant analysis (LDA) are used to highlight molecular mechanisms that discriminate different classes of samples.

Many variations of PLS, a common dimensionality reduction method, have been introduced for the integration of complex datasets: for example, Integromics [3] relies on a sparse version of PLS (sPLS), and other variants of PLS, such as Orthogonal PLS [22], Kernel PLS [23] or O2-PLS [24], have been described in the literature. The idea of weighting the behavior of a gene at different levels and then combining such weights in order to get an integrated picture, applied so far for gene expression, CNV and methylation data [19], is a versatile approach that can be applicable to other types of datasets (e.g. gene expression, somatic mutations and protein expression). Thus, below we will describe in more detail Partial Least Squares (PLS) and the use of signal-to-noise statistics for the integrative analysis of multiple datasets [19].

#### Partial least squares

PLS and PCA (Principal Component Analysis) are techniques that seek to identify a small set of features that work as predictors of the response dataset. While PCA works in a purely unsupervised fashion, PLS makes use of the response in order to find appropriate linear combinations of the predictors that define a new set of features. In PLS the coefficients of the linear combination are chosen so that the highest weight is assigned to variables that are most strongly correlated to the response. In this sense we can say that PLS is a supervised alternative to PCA, for details see [25].

Multi-block PLS [4] is a method for performing PLS on a multi-layered dataset. Like any supervised PLS regression problem, sMBPLS’s set up consists of *n* (e.g. *n*=3) input layers *X*_1_,*X*_2_,*X*_3_ and a response dataset *Y*, where observations are made on the same set of samples. The goal is to identify MDRMs (Multi dimensional regulatory modules) that are column subsets of the input datasets on the same samples that are strongly associated to the response. First each layer is represented as the first PLS predictor for *i*=1,2,3, (**Z**_*i*_=*X*_*i*_·**w**_*i*_) and the response *Y* is treated the same way (**U**=*Y*·**v**), where **w**_*i*_, **v** are the loadings and **Z**_*i*_ and **U** are the summary vectors or latent variables of respectively the input and response datasets. Then sMBPLS defines **Z**=*b*_1_**Z**_1_+*b*_2_**Z**_2_+*b*_3_**Z**_3_ that is a summary vector of the three datasets. The weights *b*_*i*_ are supposed to account for the contribution of the *i*-th dataset to the total covariance. Mathematically the problem can be described as finding the optimal parameters so that the covariance between input and response (summarized in **Z** and **U**) is optimized. The results improve substantially by introducing a constraint or a penalization to the objective function that needs to be optimized: sMBPLS uses a Lasso penalization - many different penalization choices are possible (for details see e.g. [25]). The effect of this penalization is often called sparsity, meaning that negligible coefficients tend to be drawn to zero. So the final function to be maximized can be expressed as

(1) $$\begin{array}{@{}rcl@{}} \Omega(\mathbf{Z},\mathbf{U},\mathbf{w}_{i},\mathbf{v},\mathbf{b})={cov}(\mathbf{Z},\mathbf{U}) - \sum_{i=1}^{3} \mathbf{P}_{\lambda_{i}}(\mathbf{w}_{i}) - \mathbf{P}_{\lambda_{4}}(\mathbf{v}) \end{array} $$

with the further restrictions that vectors **w**_*i*_,**v**,**b** must have norm equal to 1; here $P_{\lambda _{i}}$ are the Lasso penalizations. In order to estimate the optimal parameters in (1) Li et al. develop an ad hoc algorithm [4].

#### Gene-wise weights

Multi-omics gene-wise weights have been proposed to fuse three types of omics into a unique summary score for each gene [19]. These scores *s*_*i*_ are defined using gene expression, DNA methylation and CNV data:

(2) $$\begin{array}{@{}rcl@{}} s_{i}=\left(w_{i}^{me}+w_{i}^{cn}\right)\cdot \epsilon_{i}, \end{array} $$

where $w_{i}^{me}$ and $w_{i}^{cn}$ are measures of the expression difference of the *i*-th gene between samples with high and low values of DNA methylation $w_{i}^{me}$ and CNV $w_{i}^{cn}$, while *ε*_*i*_ is a normalization term. More precisely, layer-specific weights for each gene are calculated using the mean and standard deviation of gene expression

(3) $$\begin{array}{@{}rcl@{}} w_{i}=\frac{m_{i,1}-m_{i,0}}{\sigma_{i,1}+\sigma_{i,0}}, \end{array} $$

where the suffixes 1 and 0 indicate, respectively, samples having high and low values of the other omics (DNA methylation or CNV). In summary, each variable is associated with the sum of a set of signal-to-noise scores, each of which is calculated considering the means and standard deviations of the variable using two subsets of samples of a given dataset (e.g. gene expression) defined on the basis of the values of the same variable in another layer (e.g. CNV or methylation).

### Network-free bayesian (NF-BY)

Parametric or “strict” bayesian frameworks assume that the prior probability distribution follows a specific model dependent on one or more parameters. If the prior fits the data well parametric bayesian methods usually outperform non-parametric ones. On the other hand, if the initial guess for the prior is hard or even impossible to formalize, non-parametric or distribution-free methods are preferred [7]. It is important to remark that non-parametric or distribution-free methods are characterized by the fact that - unlike their parametric counterpart - the priors are not identifiable with a given family of probability distributions depending on one or more parameters, since this family would be too large, therefore introducing the need of an alternative definition of the priors in which - roughly speaking - the parameters themselves are supposed to be random. In this context, Antoniak [26] defined Mixtures of Dirichelet Processes (DPM) a useful set of priors for many non-parametric problems, that was taken as a starting point for many recent works aiming at the integration of multi-omics, such as TMD [27], MDI [28], PSFD [29], while, for example, iCluster [12] is a parametric method. The choice between parametric and non-parametric models is often not arbitrary, but it is driven by the type of data to be modeled.

iCluster [12] and MDI [28] have been developed with the main objective of sample clustering and can be applied to different types of omics. iCluster [12] takes as input two or more matrices and finds multi-omics clusters jointly estimating, by means of a prior-posterior bayesian structure, the clustering *Z*, which is modeled as a Gaussian latent variable having layer-specific weights and parameters. MDI (multiple dataset integration) [28] carries out the same objective (clustering) using a bayesian approach to jointly estimate the parameters of Dirichelet Process Mixture models. These models are applied to find clusters and relevant genes (features).

An approach closely related to MDI is Savage’s Transcriptional Modules Discovery (TMD) [27] who also adopts a mixture modeling approach, using hierarchical Dirichelet process to perform integrative modeling of two datasets. Conversely to MDI, TMD aims at the identification of molecular mechanisms.

Patient-Specific Data Fusion (PSDF) [29] extends the TMD model for assessing the concordance of biological signals of samples in the two datasets taken into account (CNV and gene expression data). PSDF can be used to shed light on molecular mechanisms and cluster samples.

Coalesce [30] is a combinatorial algorithm specifically developed for the identification of regulatory modules from the analysis of gene expression and DNA sequence data. The multi-omics probability for a gene to be included into a module is calculated combining omics specific probabilities through the Bayes’ rule.

Since iCluster was introduced, it is often being cited by subsequent works as an innovative reference approach for multi-omics clustering of samples, while, as already said, MDI shares a multi-layer analysis approach (based on Dirichelet Process Mixture models) with other recent methods. Hence, we will focus on iCluster and MDI in the following.

#### Bayesian latent variable models

In 2009, Shen et al. developed a joint variable model for integrative clustering, naming the resulting methodology iCluster [12]. Considering *N* datasets referred to the same group of samples, iCluster formulates sample clustering as a joint latent variable that needs to be simultaneously estimated from multiple genomic data types. The first step is to capture the similarities among genomic information in each data set, so that the within-cluster variance is minimized. This task is performed by an optimization through PCA of the classical *K*-means clustering algorithm, with the additional advantage of reducing the dimensionality of the data: if *k* is the number of clusters, the dimensionality *n* of the genomic data is basically reduced to the first *k*-1 principal directions. Second, the clustering scheme in each layer is represented as a Gaussian latent variable model with the Gaussian latent component *Z* capturing the dependencies across the data types. Dealing with *N* different omics measurements on the same *p* samples *X*_1_,*X*_2_,…,*X*_*N*_, each one of dimension *p*×*n*_*i*_ with usually *p*<<*n*_*i*_, the model can be written in the following fashion:

(4) $$\begin{array}{@{}rcl@{}} X_{i} & = & W_{i} \cdot Z + \epsilon_{i} \end{array} $$

where the matrices *W*_*i*_ are the *p*×*k*−1 weight matrices and *ε*_*i*_ are the independent error terms. After taking a continuous parametrization *Z*^∗^ of *Z* and assuming *Z*^∗^∼*N*(0,*I*) and *ε*=(*ε*_1_,…,*ε*_*N*_)∼*N*(0,*cov*(*ε*)), likelihood-based inference is obtained through the Expectation-Maximization (EM) algorithm [31]. iCluster requires the number of desired clusters *k* as input for the algorithm.

Recently, Kirk et al. [28] presented a bayesian method for the unsupervised integrative modeling of multiple datasets. MDI integrates information from a wide range of different datasets and data types simultaneously. In a general *N*-components mixture model, the probability density for the data *p*(*X*) is modeled using Dirichelet-multinomial allocation mixture model,

(5) $$\begin{array}{@{}rcl@{}} p(X) = \sum_{k=1}^{N} w_{k} \cdot \pi(X|\theta_{k}) \end{array} $$

where *w*_*k*_ are the mixture proportions, *θ*_*k*_ are the parameters associated to the *k*-th component and *π* is a parametric density. Component allocation variables and some additional parameters - conversely from the TMD model [27] - are introduced in order to capture the dependencies among these models and find clusters of genomic entities having the same behavior in different datasets. The modeling structure of the multi-layer dataset exploits the mathematical connection between mixture models and Dirichelet Processes, a non-trivial problem: for details see [32]. In this way is possible to construct a prior probability for each dataset where the probability distribution is parametrized by component allocation variables. Inference on such parameters is performed through Gibbs sampling. Finally, in order to identify groups that tend to cluster together in multiple datasets, it is natural to exploit the posterior probability as a metric in order to decide whether or not a connection among each couple of genes is strong enough across the dataset.

Both MDI and iCluster carry out simultaneous integrative clustering of multiple omics datasets. However, in contrast to MDI, iCluster seeks to find a single common clustering structure for all datasets.

### Network-based non-bayesian (NB-NBY)

Methods that we have assigned to this category make either use of molecular interaction data or use networks defined from correlation analysis.

SteinerNet [33], the method proposed by Mosca et al. [16], stSVM [34] and nuChart [35] share a common strategy: the analysis of a multi-weighted graph that carry multi-omics information. SteinerNet [33] is a method that identifies molecular sub-networks using omics datasets and a given molecular network. In order to reconstruct response pathways, SteinerNet finds a solution to the prize-collecting Steiner tree (PCST) problem, a minimum-weighted subtree that find an optimal network subject to weights assigned to vertexes and edges on the basis of input datasets. Similarly, multi-objective optimization (MOO) has been recently proposed for the extraction of sub-networks enriched in multi-omics information [16]. Sub-networks are extracted on the basis of multiple criteria applied to a network that encodes several layers of biological information as vertex and edge weights. Also stSVM (smoothed *t*-statistic support vector machine) method [34] loads gene-wise statistics from multiple omics (miRNA and mRNA) on a molecular network known *a priori*. Then, a network diffusion method is used to smooth the statistics according to network topology. Significant genes are then used to train a classifier (a SVM) that predicts the type of sample (e.g. early versus late disease relapse). NuChart [35] is a method for the annotation and statistical analysis of a list of genes with information relying on Hi-C data (genome-wide data of chromosomal interactions [36]). NuChart identifies Hi-C fragments by means of DNA sequencing data and creates gene-centric neighborhood graphs on which other omics data (e.g. gene expression) are mapped and jointly analyzed.

ENDEAVOUR [37] calculates gene-wise statistics from heterogeneous genome-wide data sources (including molecular interactions) and ranks genes according to their similarity to known genes involved in the biological process under analysis. Single layer prioritizations are then integrated into a global ranking by means of order statistics. In 2007 De Bie et al. [18] proposed a kernel-based data fusion method for gene prioritization, which operates in the same setting of ENDEAVOUR. Kernels representing gene information in each layer are linearly combined in order to fuse the information and identify disease genes.

SNF (Similarity Network Fusion) [17] is a method that computes and fuses patient similarity networks obtained from each omics separately, in order to find disease subtypes and predict phenotypes. Conversely from the other methods of this section, SNF uses sample-sample networks obtained from correlation analysis. The key step of SNF is to iteratively and simultaneously update the global patient similarity matrix of each layer using a local *K*-nearest neighbours (KNN) approach combined with the global similarity matrices of the other layers. Fusion is then completed by averaging the similarity matrices once the iterative upgrading is performed.

Recently, a type of multi-partite network (multiplex) has been introduced as a novel theoretical framework for network-based multi-layer integrative analysis [38]. Multiplex networks are multi-layer systems of vertexes that can be linked in multiple interacting and co-evolving layers. This approach has been proposed for the analysis of gene expression data in brain [39] and cancer [40]. In the second example, a sample-sample duplex (two-layers network) has been generated based on correlation between gene expression profiles, revealing structural similarities and differences between two classes of samples. Thanks to their general formalism, in principle multiplex networks can be applied to the joint analysis of several types of omics (e.g. one type of omics for each layer), also for multi-level clustering purposes [41].

In the following subsections, we will discuss in more detail network diffusion, fusion of similarity networks and heterogeneous/multiplex networks. Methods that simulate the diffusion of information throughout a network are being increasingly used, since they allow to study how the information (e.g. differential expression, sequence variations) initially available in one or more network components (vertexes) affects other network regions [42]. SNF [17] is a diffusion-based strategy that can be easily extended to the analysis of a wide range of multi-omics data. Heterogeneous and multiplex networks are promising frameworks for innovative multi-omics data analysis.

#### Diffusion processes on networks

Network diffusion algorithms define a vector of scores *σ* associated with network vertexes on the basis of initial conditions **x**_0_ and network topology *τ*, usually represented by the adjacency matrix *A* or the Laplacian matrix *L* of the graph.

An application of such techniques is found in stSVM [34], where a *p*-step random walk kernel *K* is used in order to smooth the *t*-statistics **x**_0_, which assess the differential expression of genes. The kernel is defined as

(6) $$\begin{array}{@{}rcl@{}} K=(\alpha\cdot I- L^{\prime})^{p} \end{array} $$

where *α* is a constant, *L*^′^ is the symmetrically normalized Laplacian matrix of the graph and *p* is the number of random walk steps. The smoothing of the *t*-statistic **x** is simply computed using the kernel *K*:

(7) $$\begin{array}{@{}rcl@{}} \mathbf{x}=\mathbf{x}_{0}^{T}\cdot K \end{array} $$

In this case the influence of a node on the network is controlled by the parameter *p*. Basically, the information initially available in each vertex is distributed to its neighbors by means of the application of *K*. For a deeper insight of diffusion kernels see [43].

In other diffusion models, the network-based scores *σ*=*σ*(*X*_0_,*τ*) are the steady state solution of a discrete or continuous diffusion process on the network that can have either a deterministic or a stochastic interpretation. An example of such a technique is the network propagation algorithm exploited in the work of Hofree et al. [44]: after mapping a patient mutation profile onto a molecular network, network propagation is used to “smooth” the mutation signal across the network. Network propagation uses a process that simulates a random walk on a network with restarts according to the function:

(8) $$\begin{array}{@{}rcl@{}} \mathbf{x}(t)=\alpha A^{\prime} \cdot \mathbf{x}(t)+(1-\alpha)\mathbf{x}_{0},  \end{array} $$

where **x**_0_ is a vector representing some kind of genomic information about a patient (in this case mutation signal), *A*^′^ is the symmetrically normalized adjacency matrix capturing correlations among genes, and *α*∈(0,1) controls how much information is retained in the nodes with respect to how much is not. For *t*→*∞* for each patient, the discrete array **x**_0_ is smoothed into a real-valued array *σ*=**x**(*∞*).

Network diffusion processes are often based on an actual physical model, having the benefit of exploiting physical quantities and concepts to drive the setting of the parameters. For example Vandin and Upfal [45] presented a computationally efficient strategy for the identification of sub-networks considering the hydrodynamic model introduced by Qi et al. [46]: fluid is pumped into the source node *s* at a constant rate, diffuses through the graph along the edges, and is lost from each node at a constant first-order rate until a steady-flow solution is reached.

The presence of random walks on a graph allows connections to many other physical models. For example, another interesting framework is represented by electric circuits [47], where the relation between the random walk of electrons on a circuit and Kirkhoff laws is exploited. eQed is a recent application of the latter [48]. Recently Mirzaev and Gunawardena have collected and rigorously demonstrated some of the most important mathematical results in the context of information dynamics in a linear framework, also suggesting a possible stochastic interpretation of such diffusion processes on the network in the Chemical Master Equation formalism [49].

#### Fusion of similarity networks

An interesting strategy to perform simultaneous network-based integration of omics is the one at the basis of SNF [17]. A number *N* of different patient similarity networks with associated global similarity matrices *P*_*i*,0_ are defined from *N* datasets. Let’s assume *N*=2 for the sake of clarity. Then, for each layer a KNN local similarity matrix *S*_*i*_ is introduced in order to retain only robust information. Subsequently, global similarity matrices are smoothed by two parallel interchanging diffusion processes that consist of the upgrading of the global similarity matrices with respect to the local similarity matrices of the other layer:

(9) $$\begin{array}{@{}rcl@{}} P_{1}(t+1) & = & S_{1}\cdot P_{2}(t)\cdot {S_{1}^{T}}  \\ P_{2}(t+1) & = & S_{2}\cdot P_{1}(t)\cdot {S_{2}^{T}} \end{array} $$

having initial condition *P*_*i*_(0)=*P*_*i*,0_. After convergence, the fused similarity matrix is then defined as the average of *P*_1_ and *P*_2_. The result is a similarity matrix that can be viewed as the weighted adjacency matrix of a network built by fusing the similarity networks associated with each layer [17].

#### Heterogeneous networks and multiplex

In the context of multi-omics data analyses, multiple (*k*) layers can be represented by means of *k* networks. In this context, we can distinguish between two kinds of formalism: heterogeneous networks and multiplex networks.

Heterogeneous networks consider *k* different kinds of nodes, each type corresponding to a different layer of biological information. In this framework, intra-layer connections and inter-layer connections are formally treated in the same way, even if they can be weighed differently. The multi-layered information is therefore somehow squeezed on just one dimension and the properties of the resulting graph can be used to manipulate the data. For example, for *k*=2 we can have vertexes of genes layer *g*_1_,*g*_2_,…,*g*_*n*_ and proteins layer *p*_1_,*p*_2_,…,*p*_*m*_. The Laplacian matrix of this heterogeneous network is a (*n*+*m*)×(*n*+*m*) matrix:

(10) $$ L_{gp}=\left[ \begin{array}{ c c } L_{g} & B_{gp} \\ B_{pg} & L_{p} \end{array} \right],   $$

where *L*_*g*_ and *L*_*p*_ are the Laplacian matrices of respectively gene and protein layers, while the matrices *B*_*gp*_ and *B*_*pg*_ contain the information about inter-layer connections; in the case the graph is undirected $B_{\textit {pg}}=B_{\textit {gp}}^{T}$. An example of application of heterogeneous network for modeling gene-phenotype networks was presented by Li and Patra [50].

Multiplex networks [38] are instead multi-partite networks in which each of the *k* layers models a different information about the same set of vertexes *v*_1_,*v*_2_,…,*v*_*n*_. For example, let us consider two omics, represented as a two-layered multiplex composed of two sample × sample networks, where the edges of each network are placed in function of the sample-sample correlations found in the associated omics. Then, it is possible to analyze inter-layer correlations by means of multilnks, a quantity that summarizes the connectivity of each pair of samples across the layers. More precisely, a multilink is a *k*-dimensional binary array whose *i*-th component is set to 1 if the two samples are connected in the *i*-th layer and 0 otherwise. The formalism of multilink is the basis to define weighted measures and overlaps of the multiplex networks and other physical quantities, such as entropy, which introduces a theoretical framework to quantify and detect the information stored in complex networks [38, 40].

### Network-based bayesian (NB-BY)

In this section we deal with methods that can be classified as both network-based and bayesian; these features select mainly those methods that are somehow related to bayesian networks (BNs). BNs are probabilistic models composed of a graph and a local probability model that can be either parametric or not. BNs represent an important area of machine learning theory and many applications of this topic are found in diverse fields. BNs can be thought as a combination of network theory and probability theory.

Within the BN framework an important method for multi-omics data integration is Paradigm [51]. Its goal is the definition of patient-specific pathway activities by means of probabilistic inference. Each biological entity (gene, protein, etc.) is modeled as a factor graph that can be defined to host a wide range of multi-omics information, and is associated with a prior probability of being activated in a given pathway.

Conexic, a bayesian network-based algorithm, has been introduced for the identification of driver mutations in cancer through the integration of gene expression and CNVs [11]. Conexic is based on a bayesian scoring function that evaluates how each candidate gene, or a combination of genes, predicts the behavior of a gene expression module across tumor samples. Networks, more precisely regression trees, are used to encode regulation programs.

Below, we will focus on the theoretical setup of the BN developed by Paradigm [51].

#### Paradigm: an application of bayesian networks

The goal of Paradigm is the definition of an entities × samples matrix called IPA (inferred pathway activity) where IPA _*ij*_ reports a score that accounts for how likely the biological entity *i* is activated/null/deactivated in sample *j*.

The model is network-based since correlations between data points are modeled as factor graphs *Φ*=(*ϕ*_1_,…,*ϕ*_*m*_) that are used for assigning a probability for the genomic entities or variables **X**=(*X*_1_,…,*X*_*n*_):

(11) $$\begin{array}{@{}rcl@{}} P_{\Phi}(\mathbf{X}) = \frac{1}{Z} \cdot \prod_{j=1}^{m} \phi_{j}(\mathbf{X}_{j})  \end{array} $$

where *Z* is a normalization constant accounting for all of the possible settings of the variables **X** and **X**_*j*_ is a set constituted by *x*_*j*_ and its “parents” *Pa*(*x*_*j*_) that are the nodes that have a link directed to *x*_*j*_ in the network. It is important to underline that the number of features *m* is much less than 2^*n*^−1 (the number of possible edges in the graph): this “sparsity” facilitates integration. In this way it is possible to assign to each gene’s *x*_*i*_ activity *a* first a prior probability distribution and then probability distribution consistent with the dataset measurements *D*:

(12) $$\begin{array}{@{}rcl@{}} P_{\Phi}(\mathbf{x}_{i}=a,D) \propto \prod_{j=1}^{m} \sum_{S\subset_{A_{i}(a)\cup D}X_{j}} \phi_{j}(S) \end{array} $$

where *Φ* is the fully specified factor graph, $S\subset _{A_{i}(a)\cup D}X_{j}$ are all the possible configurations consistent with both the dataset measurements *D* and the fact that gene *i* is activated (*A*_*i*_(*a*) is the the singleton assignment set {**x**_*i*_=*a*}); the proportionality constant is the same as Eq. (11). The junction free inference algorithm and the belief propagation algorithm are used to infer the probabilities while EM algorithm [31] is used to learn the parameters. After inference log odds of the posterior probability distribution are used to measure the activity of each gene.

## Conclusions

Methods for the analysis of multiple layers of biological information pave the way for a more comprehensive and deeper understanding of biological systems. Indeed, several authors were able to show that the integration of multi-dimensional datasets leads to better results from a statistical and a biological point of view than single layer analyses. For example, using MCD, Charj et al. [13] showed that the integration of DNA copy number, LOH, DNA methylation and gene expression data permits the identification of a higher number of DNA explained gene expression changes and a set of genes that would have been missed in standard single layer analysis; Liu et al. [21] reported an improvement in the identification of pathways and networks integrating miRNA, mRNA and proteins; Wang et al. [17] showed that their network fusion approach applied to gene expression and DNA methylation lead to clusters of patients (corresponding to cancer subtypes) with significantly different survival rates.

A better understanding of the algorithms underlying integrative approaches is important for their correct application and further development. Network-based approaches use graphs for modeling and analyzing relationships among variables and are one of the most important classes of multi-omics methods. These approaches take advantage of algorithms for graph analysis. In particular, algorithms that propagate information on networks are being proposed in several applications and are often related to actual physical models. Networks allow to model the intricate cell’s wiring diagram and to use it as a framework for the integrated analysis of layers of biological information. However the incompleteness of experimentally detected molecular interactions is still a significant limit. Further, better tools of analysis are required, because assumptions like normality and variable independence are often not fulfilled [5]. Multi-layer network-based frameworks, such as heterogeneous and multiplex networks, allow the definition of novel tools for the integration of omics. For example, the already mentioned methods of network diffusion can be extended to such frameworks in order to get multi-omics propagation scores, and new clustering algorithms could be developed based on these multi-layer relationships. Moreover, multiple omics data can be naturally embedded in a heterogeneous network framework, for example metabolomics and genomics data, considering genes that codify for enzymes as inter-layer links, and intra-layer relationship given by *a priori* biological knowledge (like protein-protein interaction network) or by network reconstruction based on metabolomics and transcriptomics data.

Another class of interesting approaches relies on Bayes’ rule. Multilevel bayesian models (parametric or not) are facing the multi-omics challenge by building frameworks that facilitate a biologically appropriate formalism for the assumptions on the prior distribution (e.g. factor graphs, mixture models) and by programming non-trivial and efficient algorithms for parameter estimation. Assuming the bayesian framework is an interesting choice because it reduces the integration to the estimate of a smaller set of parameters, simultaneously suggesting a clear integration scheme. A limitation of such models is that for parametric methods the output strongly depends on how well the prior distribution assumption is able to capture the core information of the given dataset. Distribution-free approaches do not have such a problem but sometimes tend to lack in accuracy. In the network-based context the application of bayesian networks represents an interesting compromise between networks and probability theory. The bayesian framework is promising also regarding the issue of noise, because errors have the possibility to be formally taken into account from the beginning of the analysis.

Not surprisingly, genomics and transcriptomics are the two omics for which many and more established approaches of multi-layer analysis exist. However, the availability of methods that are not tailored for specific types of omics extends the applicability of integrative approaches also to omics that are still less covered by specific methods, such as proteomics, metabolomics or glycomics.

One of the main limitations of integrative approaches is related to dimensionality. In fact, if on one hand more layers correspond to a more complete picture of the biological system, on the other hand the dimensionality of the problem increases. However, *a priori* information on the relationships among the components of the biological system should help in reducing false discoveries.

Several methods are implemented using R [52], confirming the prominent role of this programming language in the analysis of biological data, and Matlab [53]. The availability of well-documented and user-friendly implementations is a crucial factor for the usability and spread of interesting methods. However, there are still several cases in which software packages are not provided.
